# Influence of Deformation and Stress between Bone and Implant from Various Bite Forces by Numerical Simulation Analysis

**DOI:** 10.1155/2017/2827953

**Published:** 2017-05-28

**Authors:** Hsin-Chung Cheng, Boe-Yu Peng, May-Show Chen, Chiung-Fang Huang, Yi Lin, Yung-Kang Shen

**Affiliations:** ^1^School of Dentistry, College of Oral Medicine, Taipei Medical University, Taipei, Taiwan; ^2^Department of Dentistry, Taipei Medical University Hospital, Taipei, Taiwan; ^3^School of Oral Hygiene, College of Oral Medicine, Taipei Medical University, Taipei, Taiwan; ^4^School of Dental Technology, College of Oral Medicine, Taipei Medical University, Taipei, Taiwan; ^5^Division of Family Dentistry, Department of Dentistry, Taipei Medical University Hospital, Taipei, Taiwan; ^6^Department of Business Administration, Takming University of Science and Technology, Taipei, Taiwan

## Abstract

Endosseous oral implant is applied for orthodontic anchorage in subjects with multiple tooth agenesis. Its effectiveness under orthodontic loading has been demonstrated clinically and experimentally. This study investigates the deformation and stress on the bone and implant for different bite forces by three-dimensional (3D) finite element (FE) methods. A numerical simulation of deformation and stress distributions around implants was used to estimate the survival life for implants. The model was applied to determine the pattern and distribution of deformations and stresses within the endosseous implant and on supporting tissues when the endosseous implant is used for orthodontic anchorage. A threaded implant was placed in an edentulous segment of a human mandible with cortical and cancellous bone. Analytical results demonstrate that maximum stresses were always located around the implant neck in marginal bone. The results also reveal that the stress for oblique force has the maximum value followed by the horizontal force; the vertical force causes the stress to have the minimum value between implant and bone. Thus, this area should be preserved clinically to maintain the structure and function of a bone implant.

## 1. Introduction

The lifetime of a buried implant is evaluated by two phases as masticatory forces are applied: the unloaded healing phase and functional phase. Implants may fail during either phase, typically for different reasons. Failure during the first phase happens within a short time after implant placement and is associated primarily with inflammation [1]. Failure during the second phase takes place after implant loading and is associated mainly with direction of a load incorrectly oriented along the long axis of an implant [2, 3]. Implant size affects the area of possible retention in bone; factors such as occlusion, masticatory force, number of implants, and implant position within prosthesis affect forces acting on bone adjacent to implants [4]. Kashi et al. [5] showed that maximum stresses happened at the location of the first screw hole (closest to the condoyle) of an implant. The highest microstrain was observed in bone adjacent to the first screw hole.

An applied mechanical force generates stress and strain in bone, deforming its structural arrangement. Van Oosterwyck et al. [6] showed that a dehiscence can happen when a narrow alveolar bone ridge is served with an oral implant. The presence of buccal and/or lingual dehiscence leads to a marked increase in marginal bone strain at the implant's mesial and distal sides. Faegh and Muftu [7] revealed that the highest continuous interfacial stresses exist in the region where an implant collar engages the cortical region and near the implant apex in the subcortical region. Detolla et al. [8] took advantage of computer-aided design (CAD) and FEA to evaluate stresses on implant surfaces and in surrounding bone. Menicucci et al. [9], who used two-dimensional (2D) and three-dimensional (3D) finite element analysis (FEA) to determine peri-implant stress occurring during tooth loading, revealed that the static load was more harmful for peri-implant bone than transitional load. Tada et al. [10] applied three-dimensional finite element analysis to evaluate the effect of implant type and length. The results demonstrated that cancellous bone of higher rather than lower density might ensure a better biomechanical environment of implant. Geng et al. [11] reviewed the application of FEA in dental implant studies. Based on clinical observations, some dentists have declared that marginal bone loss around the implant neck is approximately 0.5–1 mm or as high as 1.5 mm during the first year after implant loading. Subsequently, the loss rate of bone is considered either stable or significantly reduced (bone loss of approximately 0.1 mm), or resort of the bone crest continues and the implant is ultimately lost. These findings are in accordance with those gained 3D mathematical models of dental implants under nonaxisymmetrical loading, demonstrating that maximum stress happened around the implant neck.

This study compared the effect of various bite forces (vertical force, horizontal force, and oblique force) on deformation and stress distributions around the implant. The vertical force is the normal chewing ability (masticatory force). The horizontal force means the molar force when human being is sleeping. The oblique force is as the human being eats the irregular food (special masticatory force). Thus, single cylindrical dental implant placed vertically into the molar region of the mandible was modeled using 3D graphics. Deformation and stress distribution in the bone socket after loading by bite force in different directions was computed by FEA.

## 2. Numerical Method

**Figure 1:**
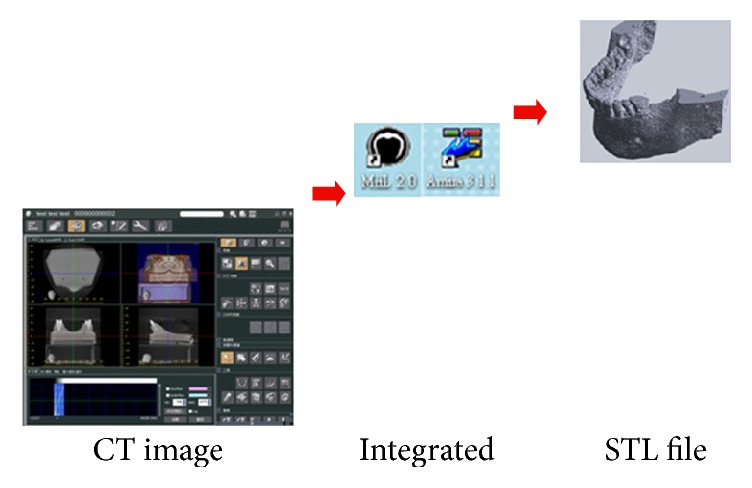
3D model creation for numerical simulation.

**Figure 2:**
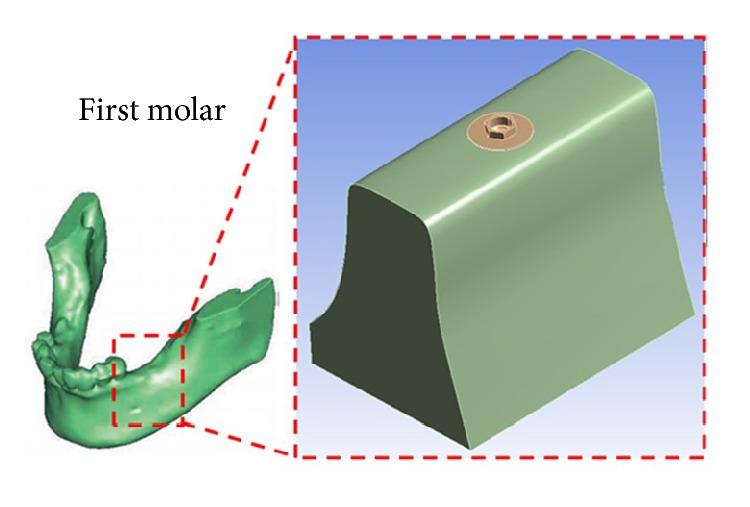
Mandible model for numerical simulation.

**Figure 3:**
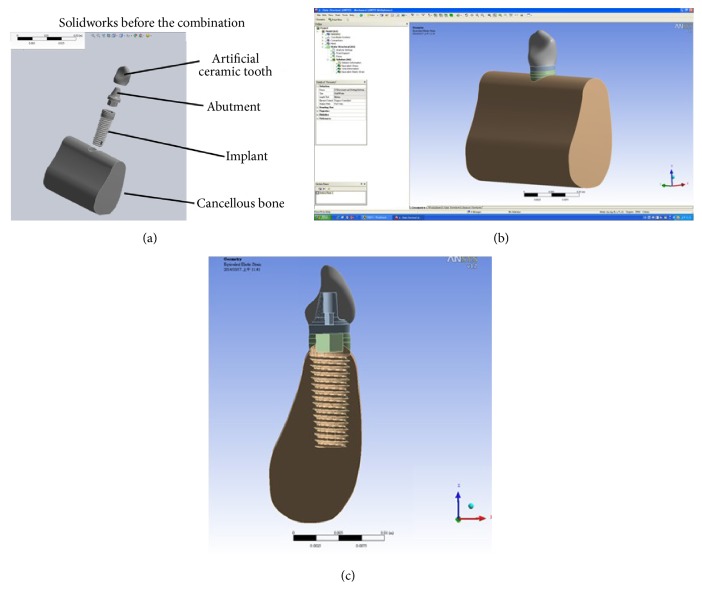
Implant system: (a) part, (b) assembly, and (c) cross section.

A computer tomography (CT) scanner of dentistry (i-CAT 3D; Imaging Sciences International, Inc., USA) was applied to scan a patient's mandible. The patient was a 35-year-old male recruited from Taipei Medical University Hospital, Taiwan. Mill and Amira V3.1.1 software program was taken advantage of to integrate the dental CT scan and 3D model. Finally, this study employs the Solidworks software to get the STL file of the 3D model. This model can be applied as the initial model for numerical simulation using ANSYS software.  Figure 1 shows the process of this schedule. Finite element analysis is commonly applied for deformation and stress analysis of nonrigid bodies. The area of interest, the model (an implant and part of a mandible list in Figure 2), is divided into element meshes. The material properties of implant system bone are shown in Table 1.  Figure 3 shows the model of implant system and cancellous bone. The physical properties of each element are fixed. Boundary conditions, restrictions to physical properties stemming from the physical behavior of the patient, are used for outer model elements. Deformation or stress, for example, was determined within each element in the model. In this study, FEA was used to analyze deformations and stresses created by different bite forces around cylindrical dental implants. Dimensions of the reference implant were selected based on those of the implant most frequently used in Taiwan (data were obtained from implant distributors).

**Figure 4:**
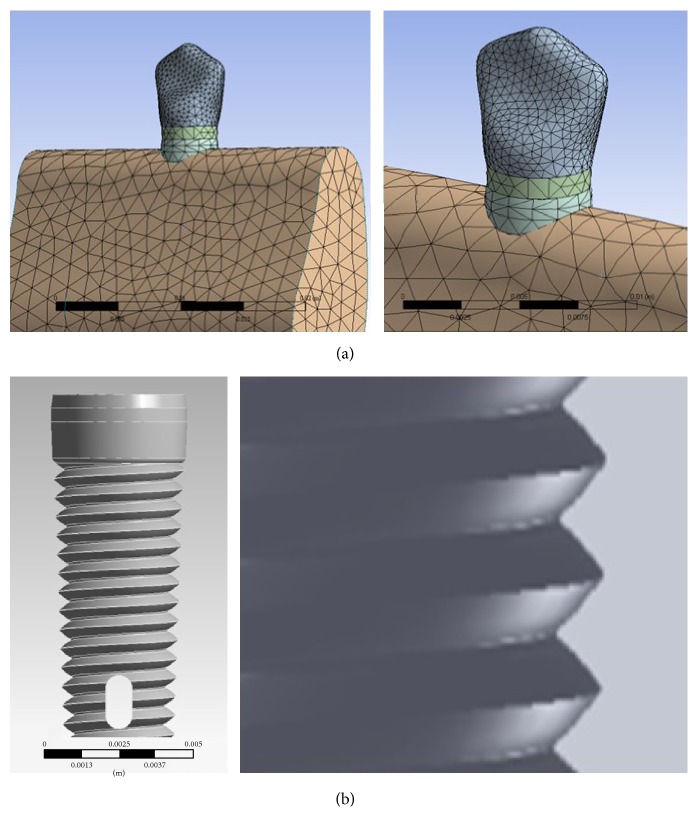
Mesh and screw type of implant system: (a) mesh and (b) screw type (diameter = 4.5 mm, pitch = 0.7 mm).

The mesial and distal borders of the end of the modeled mandible section were restricted, such that displacement of nodes in all directions was zero. The whole bone was supposed as a homogenous and isotropic material with the characteristics of cancellous bone. This was realized as spongy bone that changed its structure after successful implant placement and the implant interface changes into increasing similar to cortical bone. The tooth is zirconia material. The most frequently used implants in Taiwan are 4.5 mm in diameter, 0.7 mm in pitch, and 8 mm in length. Relying on implant size, the models consisted of 27855 elements and 47646 nodes (Figure 4).

Geometric three-dimensional models of the implant and molar region of the mandible and material properties (previously mentioned) of bone were simplified to decrease computing time and memory consumption. The authors suppose these geometric modifications do not influence computational accuracy in terms of deformation and stress distributions. The simplifications applied in this study did not affect analytical results, because all models had undergone the same simplification. Model simplification has a basis; important factors are taken into account; so the differences with the real results are not.

To yield the 3D model, pre- and postprocessor ANSYS computer-aided engineering (CAE) in FEA software (ANSYS V12) was applied. This pre- and postprocessor offers a parametric definition of geometry and the FE mesh (ANSYS, V12).

**Figure 5:**
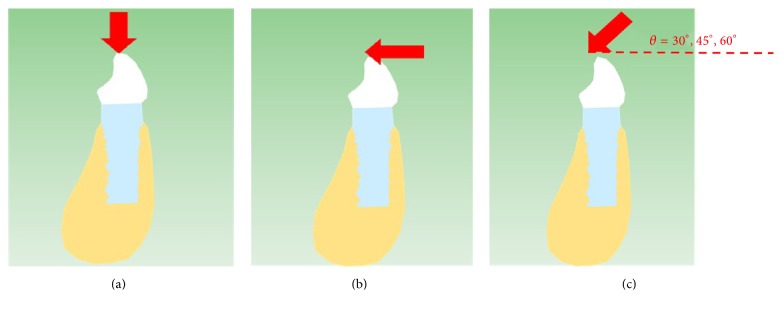
Various bite forces: (a) vertical force, (b) horizontal force, and (c) oblique force.

Loading of implants in three dimensions with various bite forces (masticatory force (vertical force), molar force (horizontal force), and special masticatory force (oblique force)) (Figure 5), the values of the bite forces used are 500 N, 1000 N, 1500 N, 2000 N, and 2500 N. The special masticatory force in different angles (30°, 45°, and 60°) is used relative to the occlusal plane. This 3D loading acted on the center of the upper surface of the tooth. Force magnitudes, as well as the acting point, were chosen based on previous work at Taipei Medical University Hospital. Assuming that the implant binds to the bone, the interfacial condition is bonded [12]. Pre- and postprocessing were conducted on a personal computer (PC). The CPU of PC had an Intel type (Core 2 Q8200) and 3.5 GB RAM. Computation time for each simplified single-size implant was approximately one and half hours. All computations were fulfilled for the 3D models. The authors had done the test of element quality; element quality is mostly close to 1. So the results are credible. The deformation and stress (Pa) at the implant-bone interface were computed using FEA software.

## 3. Results and Discussion

**Figure 6:**
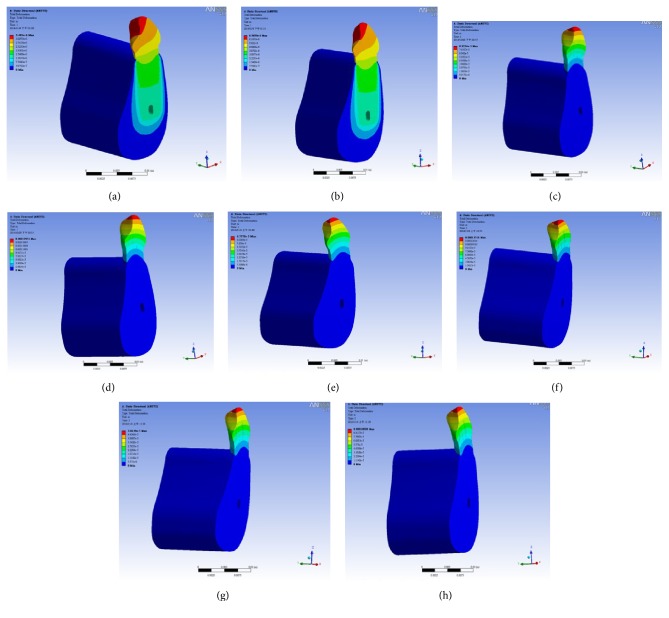
Deformation distributions for various forces: (a) 500 N, vertical force; (b) 1000 N, vertical force; (c) 500 N, horizontal force; (d) 1000 N, horizontal force; (e) 500 N, oblique force (45°); (f) 1000 N, oblique force (45°); (g) 500 N, oblique force (60°); (h) 1000 N, oblique force (60°).

**Figure 7:**
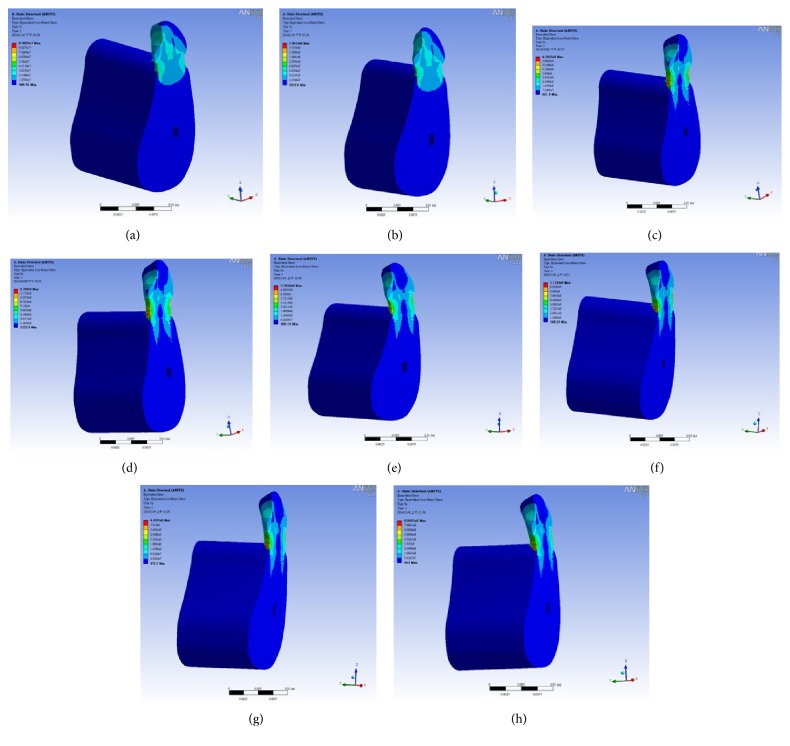
Stress distributions for various forces: (a) 500 N, vertical force; (b) 1000 N, vertical force; (c) 500 N, horizontal force; (d) 1000 N, horizontal force; (e) 500 N, oblique force (45°); (f) 1000 N, oblique force (45°); (g) 500 N, oblique force (60°); (h) 1000 N, oblique force (60°).

Figure 6 shows the deformation distributions of various masticatory forces (500 N–1000 N). Figures 6(a) and 6(b) show that the maximum deformation value occurs on the top of the tooth. Deformation value decreases as the deformation position goes to the bottom of implant. Figures 6(c) and 6(d) indicate that the maximum deformation value occurs on the top of the tooth. Deformation value decreases as the deformation position goes to the implant abutment. The deformation distribution of implant presents the symmetry by means of the central axis of the tooth. The deformation distribution of implant system by horizontal force has the difference among it by vertical force. Figures 6(e) and 6(f) reveal that the maximum deformation value takes place on the top of the tooth. Deformation value decreases as the deformation position goes to the implant abutment. The deformation distribution by oblique force (45°) shows the symmetry by means of the central axis of the tooth. The deformation distribution of implant system by vertical force demonstrates the oblique shape on the tooth. Figures 6(g) and 6(h) show that the maximum deformation value occurs on the top of the tooth. Deformation value decreases as the deformation position goes to the implant abutment. The deformation distribution of implant by oblique force (60°) presents the symmetry by means of the central axis of the tooth. The deformation distribution of implant system by oblique force (60°) has the difference among it by vertical force.  Figure 7 shows the stress distributions of various masticatory forces (500 N–1000 N). A study goal was to identify the exact location of the implant abutment. Analytical results for Figures 7(a) and 7(b) demonstrate that maximum stress was on the neck of the implant abutment. Additionally, the neck of the implant abutment was the weak point. Analytical results for Figures 7(c) and 7(d) demonstrate that maximum stress was on the neck of the implant abutment. Additionally, the neck of the implant abutment was the weak point. The stress distribution of the implant system decreases with the central axis of the implant. The stress distribution of implant system also presents decrease from neck of implant abutment to the top of the tooth. Analytical results for Figures 7(e) and 7(f) demonstrate that maximum stress was on the neck of the implant abutment. Additionally, the neck of the implant abutment was the weak point. The stress distribution of the implant system decreases with the central axis of the implant. The stress distribution of implant system also presents decrease from neck of implant abutment to the top of the tooth. Analytical results for Figures 7(g) and 7(h) indicate that maximum stress was on the abutment. A study goal was to identify the exact location of the implant abutment. Analytical results demonstrate that maximum stress was on the neck of the implant abutment. Additionally, the neck of the implant abutment was the weak point. The stress distribution of the implant system decreases with the central axis of the implant. The stress distribution of implant system also presents decrease from neck of implant abutment to the top of the tooth.

**Figure 8:**
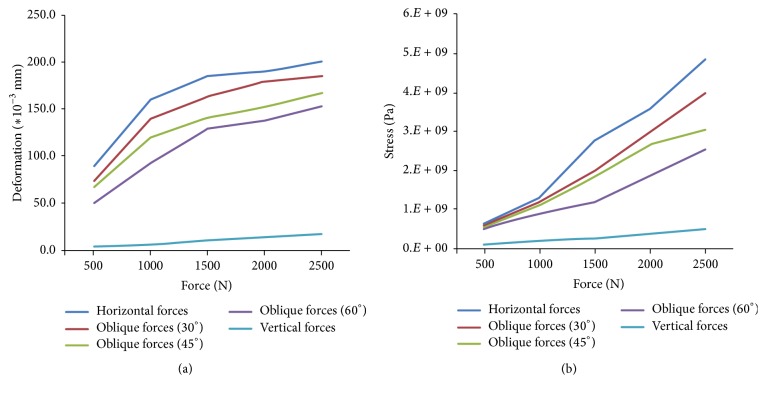
Maximum deformation and stress value on various bite forces: (a) deformation and (b) stress.

Figure 8(a) reveals the maximum deformation of implant system on different bite forces (various values). The deformation of the implant system has the maximum value by horizontal force; then it is by oblique force 30°, 45°, and 60°, and it by vertical force earns the minimum value. The maximum value of deformation increases as the bite force increases.  Figure 8(b) reveals the maximum stress of implant system on different bite forces (various values). The maximum stress value of the implant system occurs by horizontal force; then it is by oblique force 30°, 45°, and 60°; and stress by vertical force earns the minimum value. The reason is that horizontal force can generate a larger moment than other loading on the neck of the implant abutment. Vertical force acts directly on the abutment of implant. This phenomenon gets smaller moment on the implant abutment. The maximum value of stress increases as the bite force increases.

## 4. Conclusions

The deformation of the implant system has the maximum value by horizontal force, then it is by oblique force 30°, 45°, and 60°; and it by vertical force gets the minimum value. The maximum stress value of the implant system occurs by horizontal force; then it is by oblique force 30°, 45°, and 60° and stress by vertical force gets the minimum value. The deformation and stress distribution by molar force (horizontal force) show the maximum value for dental implant system. The molar force for the patient is key factor after dental implantation clinic. The results also show that the distribution of deformation and stress increases as the bite force increases. This study shows the neck of the implant system is the weak point as the bite forces come from the human being.

## Figures and Tables

**Table 1:** Material properties of implant system.

	Tooth (zirconia)	Abutment (titanium)	Implant (titanium)	Cancellous bone
Young's modules (Pa)	2 × 10^11^	9.6 × 10^10^	9.6 × 10^10^	1.37 × 10^9^
Poisson's ratio	0.3	0.3	0.3	0.3
